# Real-Time Optimization of Retinal Ganglion Cell Spatial Activity in Response to Epiretinal Stimulation

**DOI:** 10.1109/TNSRE.2021.3138297

**Published:** 2022-01-04

**Authors:** Dorsa Haji Ghaffari, Akwasi Darkwah Akwaboah, Ehsan Mirzakhalili, James D. Weiland

**Affiliations:** Department of Biomedical Engineering, University of Michigan, Ann Arbor, MI 48109 USA; Department of Biomedical Engineering, University of Michigan, Ann Arbor, MI 48109 USA.; Department of Electrical and Computer Engineering, Johns Hopkins University, Baltimore, MD 21218 USA.; Department of Biomedical Engineering, University of Michigan, Ann Arbor, MI 48109 USA; Department of Biomedical Engineering, University of Michigan, Ann Arbor, MI 48109 USA

**Keywords:** Calcium imaging, closed-loop optimization, electrical stimulation, retinal ganglion cell, retinal prostheses

## Abstract

Retinal prostheses aim to improve visual perception in patients blinded by photoreceptor degeneration. However, shape and letter perception with these devices is currently limited due to low spatial resolution. Previous research has shown the retinal ganglion cell (RGC) spatial activity and phosphene shapes can vary due to the complexity of retina structure and electrode-retina interactions. Visual percepts elicited by single electrodes differ in size and shapes for different electrodes within the same subject, resulting in interference between phosphenes and an unclear image. Prior work has shown that better patient outcomes correlate with spatially separate phosphenes. In this study we use calcium imaging, *in vitro* retina, neural networks (NN), and an optimization algorithm to demonstrate a method to iteratively search for optimal stimulation parameters that create focal RGC activation. Our findings indicate that we can converge to stimulation parameters that result in focal RGC activation by sampling less than 1/3 of the parameter space. A similar process implemented clinically can reduce time required for optimizing implant operation and enable personalized fitting of retinal prostheses.

## I. INTRODUCTION

RETINAL implants help improve functional vision for patients blinded by retinal degenerative diseases such as age-related macular degeneration and retinitis pigmentosa [1]–[3]. Percepts are created by electrically stimulating the remaining cells of the retina, including retinal ganglion cells (RGC) and bipolar cells. Patients with implants report improvements in perceiving light, detecting motion, and following lines on the ground while walking. However, their ability to recognize shapes and letters is currently limited [4], [5]. The best visual acuity is reported as 20/1260 [6] for epiretinal and 20/460 [7] for subretinal implants, both of which are lower than the acuity level for legal blindness (20/200).

The ability to precisely stimulate target neurons and avoid off-target activation is critical to create focal, non-overlapping percepts. However, human subject testing has shown that a single electrode often elicits elongated percepts [10], and *in vitro* studies have demonstrated off-target stimulation of retinal ganglion cells, confirming the clinical results [8], [9]. Unintended axonal activation is an important factor that contributes to elongated responses and low resolution of retinal stimulation. Other factors include large electrode size, electric field spread [10], [11], and spatiotemporal interactions between electrodes [12]. Prior work has related visual acuity and other visual task performance metrics with two point resolution in retinal prosthesis patients [13]. Thus, creating focal percepts is important for better patient outcomes with artificial vision systems.

Previous studies have focused on modulation of stimulation parameters to avoid axonal activation. Some of these strategies include using long duration pulses [9], and low-frequency sinusoidal stimulations [14]. While successful at avoiding axonal activation, these protocols have not proven to be feasible clinically due to high threshold charge densities associated with long pulse durations. Our previous study showed that symmetric and asymmetric anodic-first pulses with low duration ratios (ratio of anodic to cathodic phase duration) can preferentially activate RGC somas and reduce axonal activation [8]. However subsequent clinical experiments did not show significant improvement in phosphene elongation with those pulses, which may be due to the limited parameter space explored in these tests [15]. In addition to phosphene elongation, phosphene shapes and thresholds are highly inconsistent across electrodes and subjects [10]. This variability confirms that despite clinical use of retinal implants, the visual experience of patients is not adequately understood [16]. Contributing factors to these inconsistencies are variable electrode-retina separation, complex axonal pathways, heterogeneous degeneration, and perceptual interpretation of electrically elicited neural activity [17], [18]. Previous studies have shown that modifying stimulus parameters can transform the spatial RGC activity [8], and phosphene shapes [19]. Manually tuning each electrode is time consuming and tiring for patients, even when pulse shapes are limited to symmetric, biphasic pulses. Adding asymmetric pulses as an option will increase flexibility and may offer some benefits with respect to threshold and percept consistency, but this expands the parameter space to cover during a fitting procedure. Patient participation is required to confirm improvements in percept shape, but a prolonged fitting procedure will diminish the patient’s willingness and ability to provide useful feedback. Therefore, there is a crucial need to make the fitting process as efficient as possible.

Optimization algorithms have been applied to aid clinical decision making for deep brain stimulation implants [20]. In this study, we demonstrate a process that optimizes RGC spatial activity. We developed neural network (NN) models of RGC spatial activity and a real-time optimization method to search for stimulation parameters that elicit focal responses from *in vitro* retina. This work is based on our previous computational study demonstrating optimization of stimulation parameters for focal RGC activity [21]. The work presented here extends our prior work in the following ways: 1. We include in vitro retina recording as part of the optimization (not pre-recorded data) 2. We do not have prior knowledge of the response characteristics 3. NNs are created real-time 4. A convolutional neural network (CNN) is used to classify the response shape. Using this approach, we can rapidly identify stimulation parameters that produce a focal response based on sampling less than 1/3 of the possible pulse parameter combinations. A similar process may be applicable to a clinical setting for efficiently tuning phosphene shape to improve the function of visual prostheses.

## II. METHODS

### A. Overview

Wild-type mice C57BL/6 (n = 10) aged 3–4 weeks purchased from Envigo were used for calcium imaging experiments. Mice were injected with an adeno-associated virus (AAV) vector encoding a genetically encoded calcium indicator (GECI) 3 – 4 weeks prior to being euthanized for experiments. All procedures were approved by the Institutional Animal Care and Use Committee (IACUC) and the Institutional Biosafety Committee (IBC) at the University of Michigan.

### B. Intravitreal AAV Injection

To transduce the GECI jGCaMP7f in RGCs, pGP-AAV-CAG-Flex-jGCaMP7f-WPRE (Addgene #104496) was obtained from Addgene (Watertown, MA). The plasmid was then modified by the University of Michigan Vector Core to create the final vector pGP-AAV-CAG-jGCaMP7f-WPRE. Mice were anesthetized with intraperitoneal injection of ketamine (100 mg kg^−1^) and xylazine (10 mg kg^−1^). Pupils were dilated with 1% tropicamide and 2.5% phenylephrine hydrochloride. Topical tetracaine hydrochloride was applied for local anesthesia. A pilot hole was created through the sclera, choroid, and retina 1 – 2 mm posterior to the corneal limbus using a 30-gauge needle. A 5 *μ*l Hamilton syringe (Hamilton Robotics, Reno, NV) with a 32-gauge blunt needle was used to inject 1 *μ*l (1.83 × 10^12^ vg/ml) of pGP-AAV-CAG-jGCaMP7f-WPRE in the vitreous area. Injection was done slowly over 30 seconds and left in place for another 30 seconds after injection and slowly retracted to minimize leakage. Antibiotic eye ointment was used on the injection site to prevent infection.

### C. Calcium Imaging

Retinas were harvested 3–4 weeks [8] after injecting pGP-AAV-CAG-jGCaMP7f-WPRE. Animals were anesthetized with ketamine (100 mg kg^−1^) and xylazine (10 mg kg^−1^). Both eyes were enucleated and hemisected inside a perfusion chamber filled with bicarbonate-buffered Ames’ Medium (Sigma-Aldrich, St. Louis, MO). After removal of both eyes animals were euthanized by CO_2_ overdose. Dissected retina was flattened by making four cuts on the periphery. Vitreous was removed with fine forceps to ensure tight coupling between retina and the microelectrode array (MEA). The MEA formed the bottom of the perfusion chamber. Retina was then mounted on a porous membrane (cat. No. JVWP01300; Millipore) attached to a titanium ring and then placed on the transparent MEA with retinal ganglion cells facing the MEA. During the experiment, retina was superfused with bicarbonate-buffered Ames’ Medium equilibrated with 5% CO_2_ - 95% O_2_ gas, and adjusted to 280 mOsm. Solution was kept at 33°C and had a flow rate of 4 – 5 ml min^−1^. Fluorescence excitation was induced by a super bright white light emitting diode (LED). Excitation and emission light were passed through a filter set (49002 - ET - EGFP(FITC/Cy2), Chroma Technology Corp, Bellows Falls, VT) and images are captured by an electron-multiplied charge-coupled device (EMCCD) camera (iXon 897, Andor Technology, Belfast, Northern Ireland) through an Olympus UPLFLN 0.3 numerical aperture (NA) ×10 objective at 10 Hz.

### D. Electrical Stimulation

A transparent microelectrode array (MEA) constructed from glass, indium tin oxide, silicon nitride, and SU-8 epoxy photoresist was used for electrical stimulation [8]. The MEA contained 60 disk electrodes with 200 *μ*m diameter and 500 *μ*m electrode pitch. Electrical stimulus pulses were generated by the PlexStim system (Plexon Inc., Dallas, Texas) controlled by a computer software. A custom circuit board was used to relay the electrical signal to the MEA. A platinum wire placed on top of the recording chamber was used as the return electrode. Stimuli consisted of charge balanced, biphasic, anodic-first current pulses delivered at 120 Hz for 5 seconds to evoke a calcium response. Cathodic phase duration was 100 *μ*s in all experiments. Five different pulse types were used in experiments: symmetric anodic-first, asymmetric anodic-first with duration ratio of 2, 5, 10, and 20. Duration ratio is defined as the ratio of the anodic phase to cathodic phase duration. Pulse amplitude range was 20 – 110 *μ*A, or 40 – 130 *μ*A for the cathodic phase, depending on the region’s response range. The anodic phase amplitude was calculated according to the duration ratio to keep the pulse charge balanced. For duration ratios 1, 2, 5, 10, pulse amplitude was incremented by 10 *μ*A within the range stated above resulting in 10 pulse amplitudes for these four pulse types. For the duration ratio of 20, a total of 6 amplitudes were delivered, due to the stimulator resolution (1 *μ*A) limiting the possible amplitude of the longer, balancing pulse. A total of 46 pulse parameter combinations were used at each retinal region.

### E. RGC Spatial Activity Analysis

For each stimulation protocol, the fluorescence images around the active electrode were recorded at 10 fps. Images were captured for 5 seconds before and 5 seconds during electrical stimulation. The baseline image was obtained by averaging images 2 – 3 seconds after recording initiation, and the stimulation image was obtained by averaging images 2 – 3 seconds after stimulation initiation. RGC spatial activity was obtained by subtracting the baseline image from the stimulation image. The resulting calcium transient image (Δ*F*) was further normalized with respect to baseline (*F*), and a threshold was selected (Δ*F/F* > 15%) to remove noise based on the typical noise in the fluorescent signal. The shape of the RGC spatial activity (response shape) was quantified with two descriptors: activation area and eccentricity. Activation area was defined as the area of the best-fit ellipse to the RGC spatial activity, and eccentricity was defined as the ratio of the distance between the ellipse foci to its major axis length. Eccentricity values are always between 0 and 1 (0 is a circle and 1 is a line segment), and are a measure of response elongation.

### F. Optimization Pipeline Overview

**Fig. 1.**
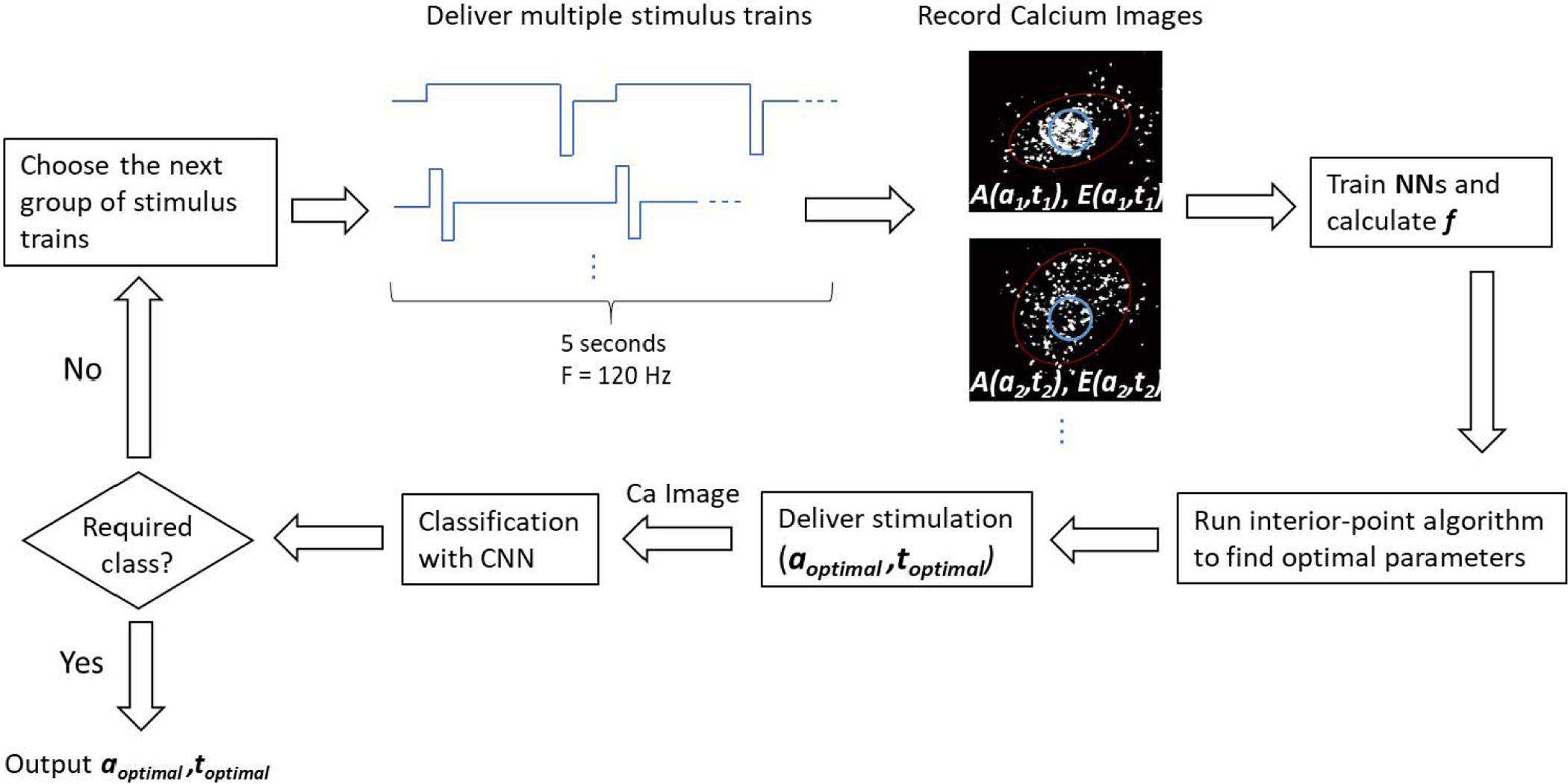
Flow chart of the optimization process. A group of 5 different stimulus trains are delivered at the beginning. Calcium images of spatial RGC activity are recorded and analyzed for area and eccentricity values. Neural networks are trained for area and eccentricity as functions of pulse amplitude and type. Interior point algorithm is run to find optimal stimulation parameters for a focal response, which is then delivered to the retina and the resulting RGC spatial activity is recorded and classified by the CNN. If the image is classified as the required class, optimal amplitude and type are reported as outputs. Otherwise, the loop continues with 10, 15, 20, and 46 different stimulus trains. Blue circles show the electrode position on calcium images, and the best fit ellipse is outlined in red.

We use artificial neural networks (NN), a convolutional neural network (CNN), and an optimization algorithm to iteratively search the parameter space and classify activation area and eccentricity, to converge to the desired response shape. Two NNs, based on images recorded during the experiment, are used to estimate surfaces for activation area and eccentricity and the resulting objective function. The optimization routine uses the objective function surface to predict optimal stimulus parameters. We record the RGC spatial activity to the predicted optimal stimulus parameters and classify the resulting image using the CNN. The procedure ends if the required class is achieved, and continues otherwise. Fig. 1 illustrates a flow chart of the optimization steps.

### G. Neural Network Training

Based on our previous results on empirical modeling of RGC spatial activity [21], a single model could not be created for the relationship between stimulus parameters (pulse amplitude and type) and the spatial response descriptors (activation area and eccentricity) that was generalizable to all regions (a region is a retinal area above and nearby an electrode). Therefore, we chose to train feedforward artificial neural networks (NN) for each region separately to quantify this relationship. Data points were divided into three subsets for training (60–80%), validation (10–20%) and test (10–20%), where the exact percentage was determined by the number of data points. The network inputs are pulse amplitude and type, and outputs are activation area and eccentricity. The NNs include a hidden layer of size 10 with hyperbolic tangent transfer functions. We used MATLAB (MathWorks, Natick, MA) built-in functions and the Levenberg-Marquardt backpropagation method for training the networks.

### H. Closed-Loop Search for Optimal Stimulation Parameters

A closed-loop optimization algorithm was developed to find stimulation parameters that elicit the desired response shape by minimizing the following objective function:

(1) f(a,t)=|A(a,t)−C|+E(a,t)

where *A* and *E* are activation area and eccentricity respectively as functions of pulse amplitude (*a*) and type (*t*), as estimated by the NNs. *C* is a constant representing the electrode area. Activation area and electrode area values were normalized to the maximum value of the activation area for a given region. The ideal response shape has an activation area equal to the electrode area and eccentricity of zero (i.e. circular).

An interior point algorithm was implemented in order to find the minimum of the objective function in each region [22]–[24]. The algorithm combines line search and trust-region steps to reduce the objective function value. At each iteration the next testing point is selected based on the direction of change in the objective function value and searching stops when the last step is smaller than the step tolerance (10^−4^). The FMINCON function from MATLAB optimization toolbox was used to implement this algorithm.

In each retinal region we started by recording the fluorescent transient images in response to 5 different sets of stimulation parameters. These points were chosen by randomly selecting one amplitude (20 – 140 *μ*A) for each pulse type. NNs for activation area and eccentricity were then trained on the images of RGC spatial activity and the objective function was created based on Eq. (1). The interior point algorithm was used to search for the minimum of the objective function and the optimal stimulation parameters. The next step was delivering a stimulus train with the predicted optimal parameters and recording the spatial RGC activity. In most cases the optimal parameters were modified to settings possible for delivery with the electrical stimulator. Similar steps were done on 10, 15, 20, (by randomly selecting 2, 3, 4 amplitudes per class) and 46 sets of stimulation parameters.

### I. Convolutional Neural Network Training for Calcium Image Classification

Prior work using a database of previously recorded RGC spatial activity images [8] showed that in most regions there were many pulse parameters combinations that resulted in a near optimal solution, and the solution found by the algorithm was not necessarily the global minimum of the objective function. Therefore, we created 5 different classes for response shape and used that as a measure of the desirability of the response shape elicited by the predicted optimal stimulus parameters. Initially, we categorized our images into 5 classes based on activation area and eccentricity values extracted from a fitted ellipse. We classified our images as class 0–4 with the following definitions: Class 0 – zero active pixels; Class 1 – eccentricity < 0.5, area < 2X electrode area; Class 2 – eccentricity > 0.5, area < then 2X electrode area, Class 3 – eccentricity < 0.5, area > 2X electrode area, Class 4 – eccentricity > 0.5, area > 2X electrode area. One metric that distinguishes different classes from each other is having an area larger or smaller than twice the electrode area. This metric was chosen because it determines whether the RGC activity overlaps with adjacent electrodes according to the electrode pitch in the MEA, which is similar to the pitch in Argus II implants. Another classification metric is having an eccentricity larger or smaller than 0.5. This number was chosen as the mid-point in the eccentricity range. However, classifying images by ellipse fitting resulted in images with sparse activity (1– 2 cells) being classified as equivalent to images with more robust activity, since ellipse fitting only required a few points. Plus, this method would classify visually similar images in different classes based on subtle differences in area and eccentricity values. Therefore, we relabeled images manually to classify them into visually distinguishable categories, and trained a convolutional neural network (CNN) for image classification. An initial total of 5466 images were labeled to distribute images into 5 classes, using our revised definition for classes 0–4: class 0 = no meaningful activity, class 1: round and small response, class 2: elongated and small response, class 3: round and large response, class 4: elongated and large response. Subsequent data augmentation to balance the number of images per class increased the total number to 8622. Data augmentation was implemented through orthogonal rotations, image flipping, and addition of gaussian and salt and pepper noise to classes 1, 2, and 3.

CNN architecture consisted of three convolutional layers each containing 128, 3 × 3 kernels and a subsequent rectified linear unit (ReLU) activation function followed by a max pooling layer (pool size = 2 × 2). The output of the convolutional layers is then flattened and fed into the dense block composed of four fully connected layers. All layers have 128 nodes, except the output layer that has 5 nodes corresponding to the five classes. The training protocol involved the use of ‘Adam’ [25] optimizer with categorical cross-entropy loss and learning rate of 0.001. A 20% dropout, L2-norm regularization (*λ* = 0.0007), and a batch size of 32 were used. Training-test data split was 90–10%, and a further 90–10% training-validation split was done on the training data. Training was done over 25 epochs while monitoring accuracy and loss performance metrics.

## III. RESULTS

### A. In Silico Prediction of Optimal Stimulus Parameters With NNs Based on RGC Spatial Activity Data

**Fig. 3.**
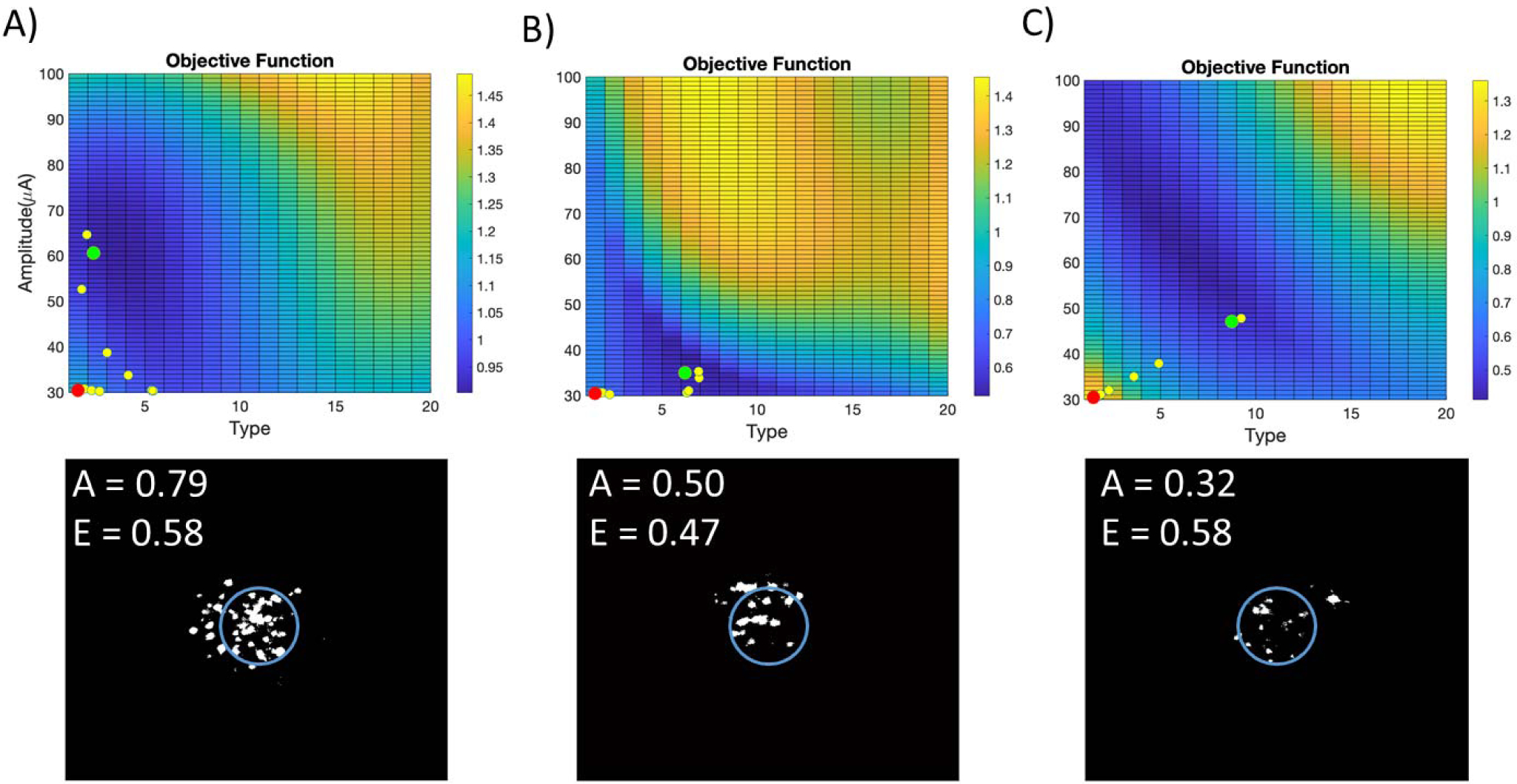
A–C) Three examples of closed-loop search for optimal stimulation parameters. Objective function maps are plotted against pulse amplitude and type. The interior point algorithm is used to search for the optimal stimulus. Red dots represent the initial condition (lowest amplitude and class), yellow dots are the intermediary points, and green dots are the optimal points. Calcium images resulting from the optimal stimulation parameters are below each objective function map. All 46 calcium images were used to create these objective functions. Normalized activation area (*A*) and eccentricity (*E*) values are displayed on each calcium image.

RGC spatial activity was obtained from 24 retinal regions during the experiments. Fig. 3A – C shows three examples of objective function maps based on pulse amplitude and type. These examples demonstrate the variability of RGC spatial activity to the same range of stimulation parameters. Stimuli that did not result in a calcium response were not included in the data points used for modeling. NNs were created for activation area (*A*) and eccentricity (*E*), and the objective function was constructed based on Eq. (1). The performance of NNs was quantified as the mean squared error (MSE) between the learned objective function maps and the experimental objective function values. The performances on the test data sets for all 24 retinal regions are shown in Table I. High standard deviation of MSE values indicates that the performance can be different for each retinal region. That is expected as it is challenging to capture the full response dynamics in some regions. The performance of NNs on training and test data indicated low overfitting. Adding more layers, nodes, or training epochs to the network can improve performance on training data but will likely cause overfitting and poor performance on test data. The stimulation and recording time for each trial took 10 seconds. Training time for NN and execution time for the interior point algorithm varied based on the amount of data and the number of iterations, respectively. But in general, these took less than 1 minute running on a standard desktop computer, without any attempt to optimize code. The operating system on the computer was Microsoft Windows 10 Pro, 64 bit, RAM = 32.0 GB, with AMD FirePro W5100 GPU. The approximate time for each number of trials in the process is below 2 minutes, 3 minutes, 3.5 minutes, 4.5 minutes, and 9 minutes, for 5, 10, 15, 20, and 46 points respectively.

Examples of interior point optimization are shown in Fig. 3. The initial condition was chosen as the point with minimum value of pulse amplitude and type. The interior point algorithm explores the continuous space fitted to the experimental data in each region, therefore the optimal point found by the algorithm was not amongst the experimental data points in most cases. Due to limitations in the resolution of the electrical stimulator, the optimal amplitudes and types were rounded to parameters within the stimulator capability. Example RGC spatial activity resulting from the closest stimulation parameters to the optimal point are shown in Fig. 3 for each corresponding objective function map. These images show relatively focal RGC activities with only sparse axonal stimulation. This confirms that the NNs and interior point algorithm are effective at finding the optimal stimulus parameters for different regions with various response characteristics.

### B. Real-Time Search for Optimal Stimulation Parameters in Vitro

**Fig. 4.**
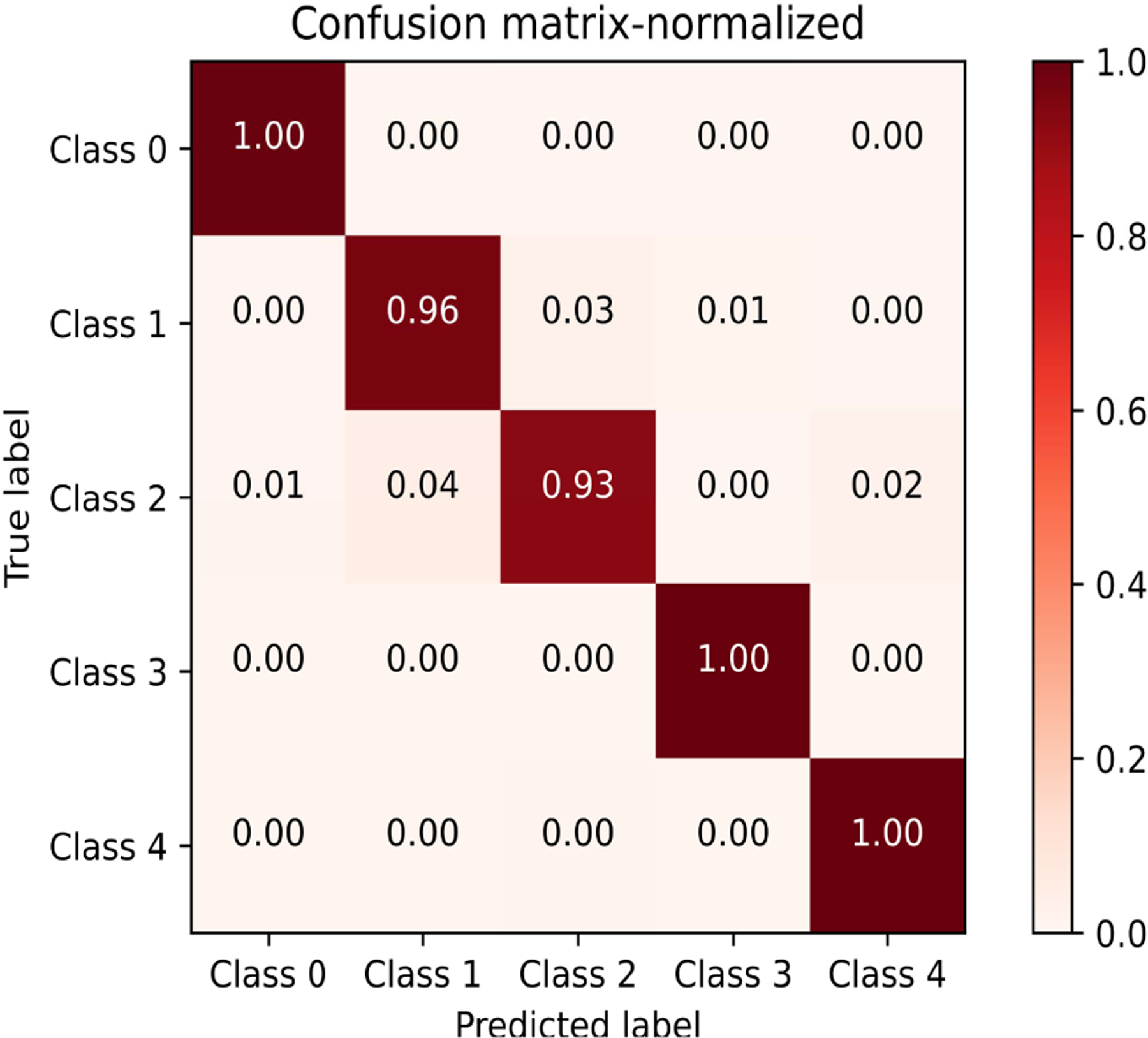
Normalized confusion matrix for CNN accuracy. Individual class recognition rates are shown for test data. Accuracy values for correctly predicting each class are shown on the diagonal.

Closed-loop optimization was performed in each region on objective function maps fitted to RGC spatial activity evoked by 5, 10, 15, 20 and 46 stimulus parameter combinations. As described in Fig. 1 and the previous section, the interior point algorithm predicted optimal stimulus parameters. Pulse parameters near the optimal settings were delivered to the retina and the RGC spatial activity was recorded. The CNN was used to classify the response shape evoked by both the randomly chosen stimulus parameters (used for NN training) and the predicted optimal stimulus parameters. The CNN performance was measured based on accuracy of predictions. Fig. 4 is a confusion matrix for true labels vs. prediction labels, showing the prediction accuracies ranging from 93 – 100%.

**Fig. 5.**
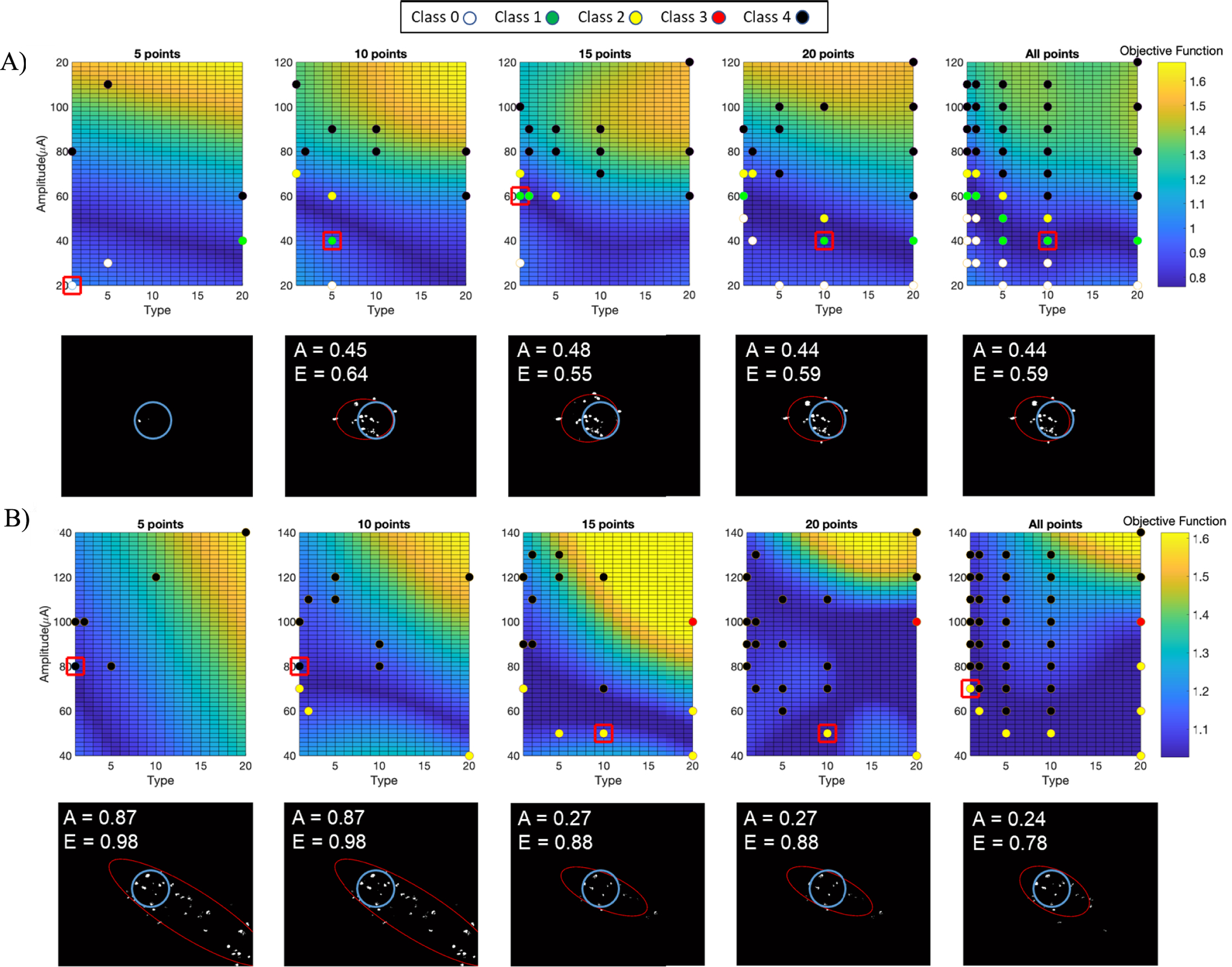
Examples for objective function maps at different iterations and the resulting optimal solution and calcium image. Colored dots are the classified calcium images for every stimulus train delivered at each iteration. Red boxes designate the optimal solution found by the algorithm. Calcium image corresponding to the optimal solution is shown below each objective function map. A) At the first iteration (5 points), the algorithm is converging to a solution with class 0 spatial activity. At iterations 2–5 the algorithm is converging to a solution with class 1 spatial activity. B) At the first and second iterations, the algorithm is converging to solutions with class 4 spatial activity. At iterations 3–5 the algorithm is converging to a class 2 spatial activity, which is the best class possible based on all trials. Normalized activation area (*A*) and eccentricity (*E*) values are displayed on each calcium image.

**Fig. 6.**
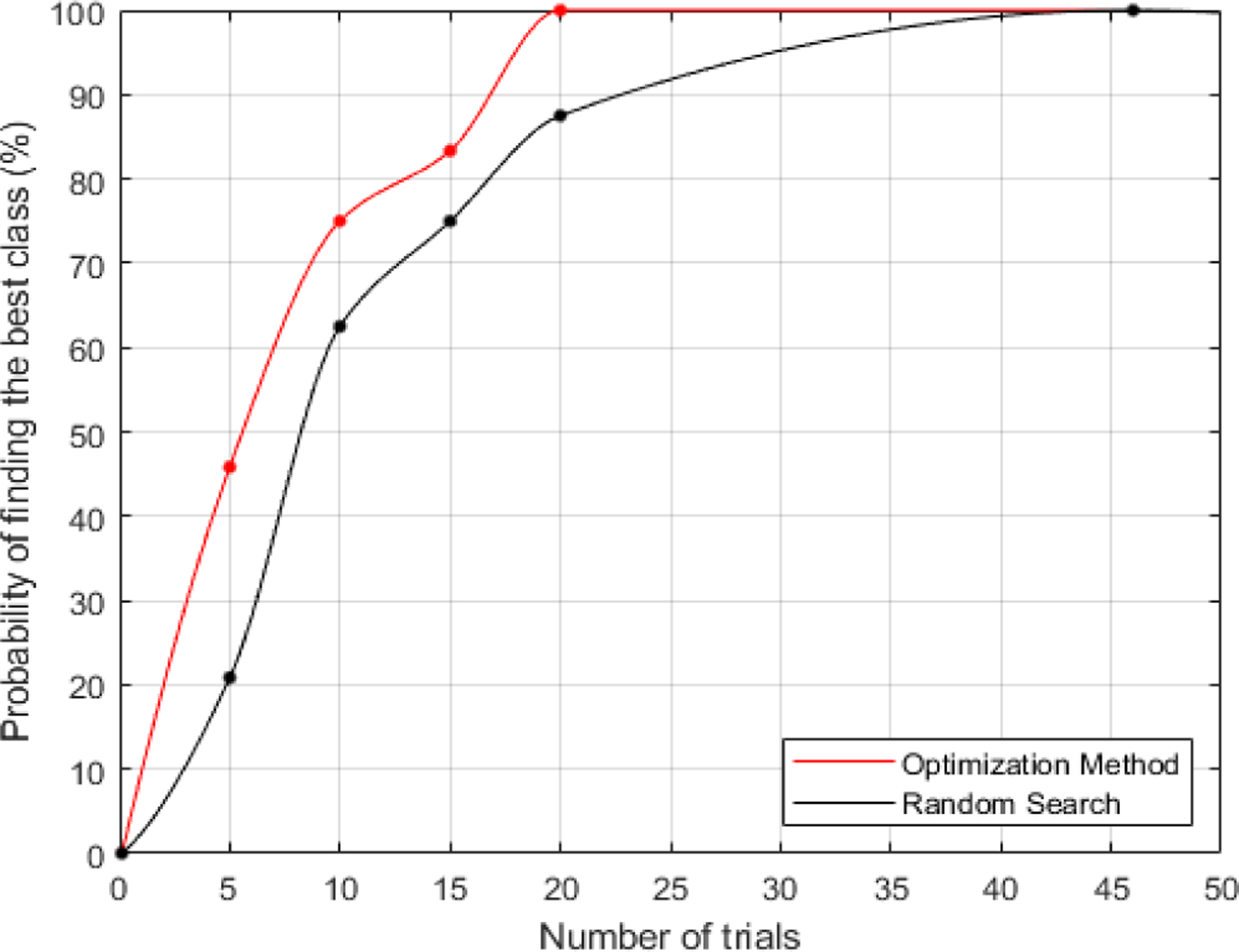
Probability of converging to the best possible class. Maximum number of trials to get the best class is 20 across all retinal regions.

The CNN classification step determined if the search was completed or if the process should continue. Since our overall goal was to create a focal response area, class 1 is the most desirable class due to focal activation area and round shape. If the CNN classified the response shape as class 1, then the process was completed. Otherwise, a new set of data was collected with more data points. Class 2, 3, and 4 follow class 1 in terms of desirability. Class 0 means no meaningful activity in response to retinal stimulation. The best class in each retinal region was defined as the most desirable class achievable considering response areas across all stimuli. Class 1 was not always achievable, but all regions yielded either a class 1 or 2 response area using this range of parameters. Class 1 was reached in 7 out of 24 regions and Class 2 was reached in 17 out of 24 regions. Fig. 5 includes examples of the optimization process in two different retinal regions. The best possible class in these regions is 1 and 2 shown in fig. 5A and fig. 5B respectively. Best class was achieved after 10 and 15 trials in these examples. Fig 6. Shows the possibility of getting best class vs. the number of trials. In all retinal regions, we achieved response shape with the best class after 20 trials. The average and median number of trials for achieving best class was 10. As a control, we randomly selected 5, 10, 15, and 20 stimulation parameter combinations, classified resulting images, and identified which groups of results had the best class image, and calculated the probability of finding the best class across 24 retinal regions. The result is shown as a black trace in Fig 6. For every number of trials, the optimization method using NNs and interior point algorithm search is providing a higher probability of finding the best class.

Figure 5 shows how the objective functions become more complex when more data is used to train the NN. Objective functions based on five points are simple, with gradients in one direction. As more data points are used to train the NN, the objective function surface becomes more complex. NNs are created based on 5, 10, 15, 20, and 46 experimental points, depending on the iteration. However, objective function maps shown in Fig. 5 have a higher resolution, obtained by calculating NN outputs with inputs of pulse amplitude (resolution of 2 *μ*A) and pulse type (resolution of 1).

## IV. DISCUSSION

We have presented a process for guided modification of epiretinal stimulation parameters to produce a focal RGC response area. Prior work in patients with Argus II retinal implants has shown the importance of increasing the focality of percepts. There has been a strong correlation between two point resolution and the grating visual acuity task; the higher the two point resolution, the better the visual acuity [13]. Further work established a link between visual acuity and performance on visually guided tasks, including line following, door finding, and letter recognition. Therefore, artificial vision can be improved by creating focal, non-overlapping percepts from individual electrodes.

We have shown that we can iteratively search and classify response areas using two NNs, a CNN, and an optimization algorithm. With this approach we were able to converge to the best possible response shape in all 24 retinal regions within 20 trials. The average number of trials needed to converge to a class 1 or 2 response shape was 10. To validate our approach, we performed a full parameter space search to identify the most desirable class possible when considering the entire parameter space. This process can reduce the exploration time significantly compared to a manual search, especially when the parameter space is large. For our experiments, we limited the free parameters to only two: pulse amplitude and pulse type. We used anodic-first pulses in this study based on our results from a previous study demonstrating anodic-first pulses elicit more focal activity and avoid axonal stimulation compared to cathodic-first stimuli [8]. Other fixed parameters included cathodic pulse width (100 *μ*s) and interphase gap (5*μ*s). Increasing the number of free parameters makes a manual process less likely to succeed. However, a large parameter space will also increase the time for a semi-automated optimization process like we demonstrate. In our process, we randomly selected pulse amplitudes for each of the five pulse types tested. If the desired class was not achieved, we randomly selected a new set of pulse parameters and increased the number of settings by five, but we did not utilize the information obtained from the prior group of settings. Adding additional samples to prior data may yield a more efficient process, since the prior data can guide the selection of the next set of parameters. We observed qualitatively that class 1 and 2 responses are above class 0, but below class 3 and 4 responses, which suggests that the optimal response is slightly above threshold. Further efficiency may be achieved by identifying key parameter settings that may have high predictive power or by focusing the search around perceptual threshold. In many cases image classes do not follow a clear trend from a class 0 to 4 by increasing pulse amplitude or type. Some regions do not show a class 1 or 3 response due to axonal activation. Plus, we tested with discrete and limited number of settings, which makes the output (response class) less continuous.

In most cases there were multiple parameter combinations that resulted in a near optimal solution. Therefore, we defined five distinct response shape classes to discretize the desirability of the solution. This approach provides the flexibility to choose any of the 5 classes as the desired outcome by modifying multiplying factors for area and eccentricity, and the *C* constant in the objective function. We did not optimize for pulse efficiency, only for shape. Other studies have focused on optimizing pulse parameters for stimulation efficacy and lower thresholds by modifying pulse duration and polarities, however these studies haven’t optimized for spatial RGC activity [8], [26], [27]. Optimizing for efficiency can be added to our framework simply by selecting the most efficient of the several pulse types that create the most focal percept class. The optimal stimulation parameters predicted by the interior point algorithm were rounded to the nearest available parameter settings. The rounded settings sometimes were less optimal (as measured by the objective function value) than the original solution.

The choice of training a CNN was based on the need for a rapid execution time and to eliminate any error in the ellipse fitting process. CNNs have recently received significant attention due to their superior performance in computer vision tasks such as image segmentation and classification [28], [29]. These deep learning models are comprised of learnable convolution filters that significantly reduce input image dimensions while preserving characteristic features necessary for good decision making by the subsequently cascaded multi-layer neural network. Our results show prediction accuracies of 93–100% and a low misclassification rate. Training curves were monitored throughout training over 25 epochs, a reasonable choice that offers a balance between extreme fitting conditions. Hyperparameters such as the learning rate, drop-out, convolution kernel sizes, depth and width of the fully connected layers were iteratively adjusted to achieve an appropriately fitted curve. However, it is important to note that an irreducible error (Bayes error rate) persists even with a sufficiently trained model. Errors of 2 classes were noted (e.g. CNN classification of 2, true label 4), but this does not indicate poor CNN performance. Instead, this reflects our definition of classes. Class 1 and 3 are round but differ in area. Classes 2 and 4 are oblong and differ in area. Therefore, a misclassification of two classes is due to an image that is in-between classes in terms of area.

The interior-point algorithm needs prior knowledge of the retinal response and local derivative information in order to select the next iterations effectively. Therefore, fitting NNs to a set of images at each iteration was necessary. Initially, we tried to use polynomial fitting of the data, since this would allow the use of calculus to obtain an optimal value. However, the NN approach provides more flexibility. Each region’s response characteristics can vary and may not follow a polynomial with a fixed order. In addition, the order of polynomial is dependent on the number of samples in each iteration. When dealing with a small sample number, the order of polynomial is limited and some non-linear responses may require a higher order polynomial to capture the response dynamics. We used a hyperbolic tangent transfer function in our NNs since it is smooth, differentiable, and works well with backpropagation approaches. Algorithms that do not require prior knowledge of the system such as evolutionary algorithms and stochastic searching can also be considered for finding the optimal solution. However, these methods require evaluating the objective function at every point in the parameter space at each iteration and generally require many iterations before converging to the optimal point [30], [31]. An alternative approach to using an optimization algorithm is performing an exhaustive grid search to find the optimal inputs; however, this approach becomes less efficient as the search space scales in both parameter range and dimension.

We demonstrated that RGC spatial activity can vary for different retinal regions in response to the same stimulation parameters. This finding in *in vitro* mouse retina confirms the previous clinical findings on the inconsistency of phosphene shapes across electrodes and subjects [16]. Future work includes performing human subject testing to verify this method. If this approach is applied in clinic, it can shorten the repetitive process of drawing phosphenes for retinal implant users compared to a manual searching approach. In place of calcium images, patient drawings [16] would be used to determine the focality of percepts. NNs can be trained on a few drawings and dynamically updated as more drawings are added. The previously trained NNs on *in vitro* data could also be used as the basis in human subject experiments. Prior studies have shown that electrical stimulation responses during *in vitro* and human subject experiments are influenced in a similar way when adjusting pulse parameters. Pulse durations longer than 20 ms induced focal RGC activity *in vitro* and round, small percepts in retinal implant users [9]. In most cases the dynamic range was narrow, as evident from Fig. 5, meaning that increasing the pulse amplitude resulted in a less desirable response shape. However, adding frequency as a variable could possibly provide a dynamic range for phosphene brightness while maintaining phosphene size [19]. An alternative method for obtaining patient feedback on optimal stimulus settings could be measuring grating acuity or coupling data driven algorithms with biophysical modeling to further refine the initial parameter settings [32]. The location of electrodes on the retina may provide some prior information that allows us to modify the optimization process. Studies have shown less elongated percept shapes happen near the fovea [16], [19]. Thus, areas near the fovea may be expected to produce class 1 responses, while areas away from fovea may produce class 2 responses at best. Building up a database of expected results based on retinal location will allow the process to run more efficiently. Given that retinal degeneration often results in rewiring of the neural retina, optimization routines will be important, since electrode location alone may not predict phosphene shape if retinal degeneration is significant, heterogeneous, and patient specific. The overall outcome of retinal prostheses can be improved by developing a clinically applicable system using the presented approach for electrode-specific optimization of stimulation parameters.

## Figures and Tables

**Fig. 2.**
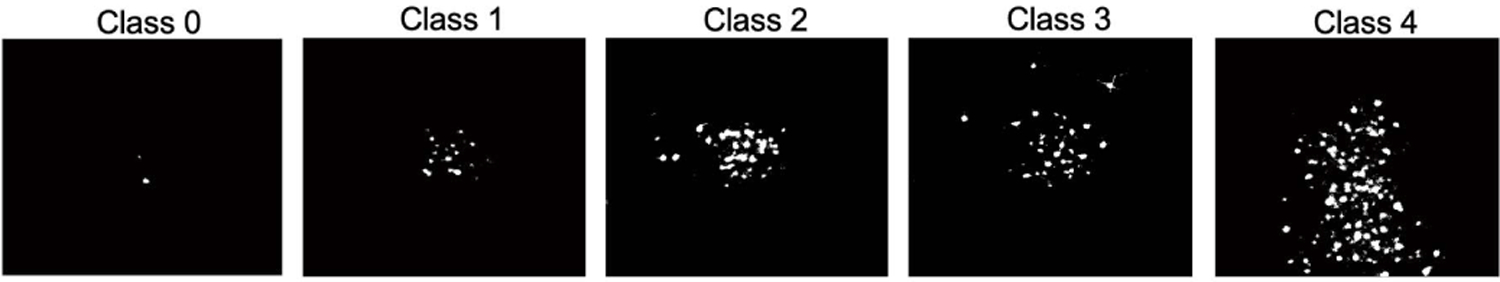
Example images for each class. Class 0: no meaningful activity, class 1: round and small response, class 2: long and small response, class 3: round and large response, class 4: long and large response.

**TABLE I:** Neural Network Performance (MSE)

	
	5 points	10 points	15 points	20 points	46 points

**Area**	0.0655±0.1068	0.0690±0.1049	0.0513±0.0858	0.0442±0.0600	0.0361±0.0394

**Eccentricity**	0.0171±0.0228	0.0191±0.0408	0.0148±0.0332	0.0130±0.0182	0.0140±0.0205
